# Thiamine Deficiency Leading to Refractory Lactic Acidosis in a Pediatric Patient

**DOI:** 10.1155/2017/5121032

**Published:** 2017-10-08

**Authors:** Alicia M. Teagarden, Brian D. Leland, Courtney M. Rowan, Riad Lutfi

**Affiliations:** Section of Pediatric Critical Care Medicine, Department of Pediatrics, Indiana University School of Medicine, Indianapolis, IN, USA

## Abstract

Thiamine plays a critical role in energy metabolism. Critically ill children and adults may develop thiamine deficiency with ultimately increased mortality due to potentially irreversible consequences of severe type B lactic acidosis. We report a case of an unvaccinated term neonate with malignant pertussis requiring extracorporeal membrane oxygenation and continuous renal replacement therapy, who developed profound lactic acidosis of unknown etiology. After countless evaluations for likely causes, the patient was ultimately determined to have thiamine deficiency and her acidosis resolved rapidly with vitamin supplementation.

## 1. Introduction

Lactic acidosis commonly results from tissue hypoperfusion and inadequate oxygenation. In patients that require extracorporeal support, tissue and end organ perfusion are constantly in jeopardy. Lactic acidosis can develop from inability to meet the body's oxygen delivery demands, with the most common causes of lactic acidosis including sepsis, bowel ischemia with associated necrosis, generalized low cardiac output, liver dysfunction, and inborn errors of metabolism. When lactic acidosis persists, other causes need to be ruled out. We report a case of an unvaccinated term neonate with malignant pertussis on venoarterial extracorporeal membrane oxygenation (VA ECMO) and continuous renal replacement therapy (CRRT), who developed refractory lactic acidosis ultimately determined to be secondary to thiamine deficiency.

## 2. Case Report

A term neonate presented to the pediatric intensive care unit with acute respiratory failure requiring invasive mechanical ventilation. Her history was significant for three days of worsening cough and apnea without associated fever, as well as an unvaccinated sibling with URI symptoms. A sepsis evaluation was initiated. A pertussis PCR was sent due to the history of apnea and unvaccinated status of a sibling and returned positive on day two of hospitalization. The patient's lung disease progressed rapidly with worsening compliance and refractory hypercarbia in association with right-sided heart failure from severe pulmonary hypertension, hemodynamic instability, and fluid overload ultimately mandating cannulation to VA ECMO support within 30 hours.

**Figure 1:**
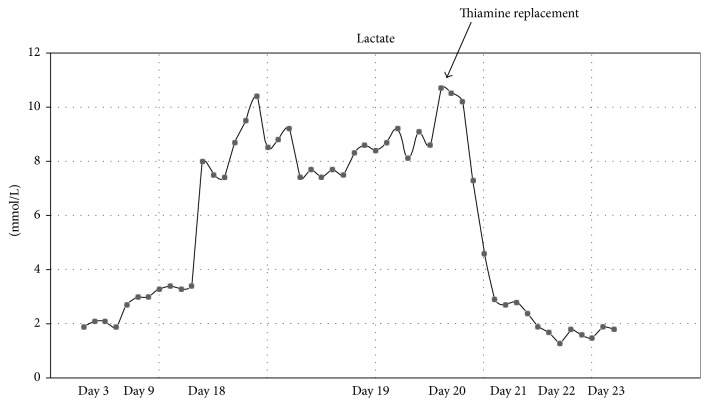
Lactate trend following initiation of thiamine replacement in our critically ill patient.

Due to concerns about abdominal distention and feeding intolerance, total parenteral nutrition (TPN) was initiated on hospital day three. A new and worsening lactic acidosis (lactate greater than 3 mmol/L and climbing) was noted by the medical team on hospital day nine, and detailed evaluation ensued. A septic workup, multiple abdominal evaluations, and attempts to clear lactate with increased ECMO circuit flows and with increased clearance using CRRT neither improved the lactate levels nor identified an etiology. Liver dysfunction and inborn errors of metabolism as possible causes of the elevated lactate were considered and ruled out. Carnitine and Co-Q10 were supplemented without apparent benefit. Lactate remained elevated for ten days to as high as 10.4 mmol/L without clear explanation. On hospital day 20, the team noted that the patient's TPN had been without standard multivitamins and trace minerals since initiation. Thiamine deficiency was proposed. A thiamine level was sent, and empiric thiamine supplementation (50 mg IV daily for 2 weeks) was initiated. Over the following 30 hours, the patient's lactate rapidly decreased from 10 mmol/L to 1.3 mmol/L, falling below 1 mmol/L by 60 hours of empiric therapy (Figure 1).

Presupplementation thiamine level returned low at 55 nmol/L (normal range 70–180 nmol/L). The patient was ultimately decannulated from ECMO support on hospital day 24 and discharged home, mechanically ventilated with a good neurologic disposition after a 94-day hospital stay.

## 3. Discussion

To our knowledge, we report the first case of an unvaccinated pediatric patient with malignant pertussis on VA ECMO and CRRT and receiving TPN, who developed severe lactic acidosis resulting from thiamine deficiency.

Thiamine, or vitamin B1, is a water-soluble vitamin that is a vital component in cellular metabolism [1, 2]. Thiamine, in the form of thiamine pyrophosphate (TPP), becomes an essential cofactor in glycolysis and mitochondrial oxidative decarboxylation of carbohydrates for energy formation [1, 3]. Diets rich in glucose, for instance, TPN, increase thiamine consumption in order to metabolize additional carbohydrate. Thiamine deficiency can lead to failure of glucose metabolism and ultimately compromise aerobic metabolism, resulting in a profound increase in the formation of pyruvate and lactic acid [2, 4].

Critically ill patients are prone to thiamine deficiency because of preexisting malnutrition, increased metabolic requirements (i.e., severe sepsis, burns, and cardiac surgery), accelerated thiamine clearance in renal replacement therapies, and increased consumption of thiamine in high carbohydrate nutrition (i.e., TPN which is often glucose-rich) [1, 4, 5]. Dietary thiamine is required daily to avoid deficiency [1, 6]. This is due to its short half-life and limited storage ability [1]. Deficiency can occur within 2–4 weeks of insufficient intake [5, 7]. Rapid recovery of thiamine levels is accomplished within hours following intravenous supplementation [4]. We observed this phenomenon in our patient when lactate levels dramatically fell after one dose of intravenous thiamine. Our patient with malignant pertussis had a number of critical interventions that increased her risk for thiamine deficiency. These included the need for ECMO and CRRT and prolonged TPN and a lack of thiamine supplementation.

To better understand the effect of thiamine deficiency, it is important to review the two types of lactic acidosis. Type A lactic acidosis is the most frequent type and commonly occurs secondary to tissue hypoxia or oxygen starvation [8, 9]. The only effective treatment for type A lactic acidosis is to improve tissue oxygenation. Therapies include augmenting cardiac output, fluid resuscitation, and treatment of sepsis. Our patient suffered from both* Bordetella pertussis* infection and respiratory stress, both of which can present with type A lactic acidosis. Type B lactic acidosis is not associated with tissue hypoxia, but rather with compromised lactate metabolism [8]. Examples include hepatic failure, defects in gluconeogenesis, or a decreased breakdown of lactate due to a deficiency in pyruvate dehydrogenase (i.e., thiamine deficiency) [8]. Our patient also developed type B lactic acidosis secondary to thiamine deficiency and worsened by high-glucose TPN. Thus, a combination of both type A and type B lactic acidosis in our critically ill patient may explain the rapid increase in lactate levels.

The underlying critical illness and clinical course placed our patient at extremely high risk of a variety of causes of lactic acidosis. Poor cardiac output, perfusion changes associated with ECMO, and the need for CRRT made the differential diagnosis and evaluation of her unexplained lactic acidosis more complicated, likely contributing to the delay in diagnosis of the thiamine deficiency.

Few studies and case reports have evaluated the negative effects of thiamine deficiency in critically ill patients. A retrospective study on critically ill adults showed that patients who progressed to death had a higher incidence of low concentrations of thiamine than did survivors [10]. A retrospective study of 11 neonates showed that infants developed severe lactic acidosis secondary to acute thiamine deficiency from TPN [11]. In fact, many of these infants had a delayed diagnosis leading to a high mortality rate. There have been few other pediatric case reports, mostly in preterm infants in the neonatal intensive care unit, revealing the serious metabolic effects from TPN-associated thiamine deficiency [9, 12].

This case discussion would not be complete without dissection of the series of errors that resulted in an iatrogenic thiamine deficiency in our patient. We offer that the thiamine deficiency in our patient likely resulted from what has become known in the literature as the Swiss-cheese model. The Swiss-cheese model describes the failure of multiple system safeguards to block errors, each of which is represented by a slice of cheese. Holes in these slices of cheese signify breakdowns in these systems that might allow errors to pass through, ultimately allowing errors to reach the patient [13]. When the TPN was first ordered for our patient, the physician unintentionally ordered it without micronutrients. The TPN was then ordered on a daily-basis as a carry-forward order in our electronic medical record, which resulted in no thiamine in the TPN for multiple days. In addition, the lack of thiamine was not noticed by other physicians, team members, and pharmacists. Thus, a learning point for providers of our patient and future patients is the need for vigilance in supplementing our critically ill patients with essential micronutrients. To our knowledge, this is the first case to report a previously undetected problem in a critically ill pediatric patient with* Bordetella pertussis* and thiamine deficiency on VA ECMO versus a consequence of an isolated series of medical mishaps.

## 4. Conclusion

Critically ill pediatric patients are at increased risk of thiamine deficiency, as described in detail above [10]. Physicians should have an understanding of these risk factors, and thiamine deficiency should be included on the differential diagnosis for persistent lactic acidosis of unclear etiology.

## Abbreviations

VA ECMO: Venoarterial extracorporeal membrane oxygenation
CRRT: Continuous renal replacement therapy
TPN: Total parenteral nutrition.
